# Tomentosin Potentiates Cisplatin-Induced Apoptosis and Modulates Proteostasis-Related Proteins in A549 Lung Cancer Cells

**DOI:** 10.3390/cimb48101015

**Published:** 2026-10-01

**Authors:** Hilal Nakkas, Beyza Ecem Oz-Bedir, Emine Terzi, Tuba Ozdemir-Sanci, Monica Angela Neblea, Tuba Aydin

**Affiliations:** 1 Department of Histology and Embryology, Faculty of Medicine, Ankara Yildirim Beyazit University, 06800 Ankara, Türkiye; hilalnakkas@aybu.edu.tr (H.N.); ozdemirsanci.tuba@aybu.edu.tr (T.O.-S.); 2 Ankara City Hospital, Ankara Yildirim Beyazit University, 06800 Ankara, Türkiye; 3 Department of Medical Biology, Faculty of Medicine, Ankara Yildirim Beyazit University, 06800 Ankara, Türkiye; beyzaecemozbedir@aybu.edu.tr (B.E.O.-B.); eterzi@aybu.edu.tr (E.T.); 4 Yenimahalle Training and Research Hospital, Ankara Yildirim Beyazit University, 06170 Ankara, Türkiye; 5 Department of Natural Sciences, Pitesti University Center, National University of Science and Technology Politechnica Bucharest, 110040 Pitesti, Romania; 6 Department of Pharmacognosy, Faculty of Pharmacy, Ağrı İbrahim Çeçen University, 04100 Agri, Türkiye; taydin@agri.edu.tr

**Keywords:** lung cancer, p62, p97/VCP, SVIP, cisplatin, tomentosin, natural product

## Abstract

Lung cancer remains a leading cause of cancer-related mortality worldwide, highlighting the need for improved therapeutic strategies and adjunct treatment approaches. In this study, we investigated the anticancer effects of tomentosin in A549 cells and evaluated the cellular response to its combined exposure with cisplatin. Cell viability was assessed using the WST-1 assay in A549 and BEAS-2B cells. Apoptosis was evaluated by Annexin V-FITC/PI staining, cell-cycle distribution by flow cytometry, and caspase-3/7 activity by a flow cytometric assay. In addition, intracellular levels of the proteostasis-related proteins p97/VCP, SVIP, and p62/SQSTM1 were quantified by flow cytometry. Tomentosin reduced A549 cell viability in a dose- and time-dependent manner, with a 24 h IC_50_ value of 24.46 ± 1.61 µM, while producing a comparatively lower cytotoxic response in BEAS-2B cells. Tomentosin increased apoptotic cell populations and altered cell-cycle distribution. Combined exposure to tomentosin and cisplatin resulted in an early apoptotic population of 72.58 ± 0.47%, compared with 2.06 ± 0.11% in control cells, and was associated with greater caspase-3/7 activity than either treatment alone. Tomentosin and/or cisplatin also produced significant alterations in p97/VCP, SVIP, and p62 levels. These findings indicate that tomentosin exerts antiproliferative and pro-apoptotic effects in A549 cells and that combined exposure with cisplatin is associated with a greater biological response under the investigated in vitro conditions. The accompanying changes in p97/VCP, SVIP, and p62 suggest modulation of proteostasis-associated cellular responses; however, their functional contribution to the treatment response remains to be established.

## 1. Introduction

Lung cancer is one of the leading causes of cancer-related mortality worldwide, with non-small cell lung cancer accounting for approximately 85% of all cases [1]. Despite current treatment options, including surgery, radiotherapy, targeted therapies, immunotherapy, and platinum-based chemotherapy, the development of chemotherapy resistance and treatment-related toxicity continues to limit therapeutic success [2,3]. Therefore, the identification of novel agents capable of enhancing treatment efficacy while reducing adverse effects remains an important priority in lung cancer research. In this context, plant-derived compounds are attracting increasing attention because of their structural diversity and their ability to simultaneously target multiple oncogenic pathways [4,5,6].

Tomentosin, a naturally occurring compound belonging to the sesquiterpene lactone class and isolated from *Dittrichia viscosa* (L.) Greuter, has gained prominence in recent years for its antiproliferative, pro-apoptotic, and cell cycle-regulating effects in various cancer cell lines [7,8]. Previous studies have shown that tomentosin can induce reactive oxygen species (ROS) generation, mitochondrial dysfunction, and activation of apoptotic cascades, suggesting that its anticancer activity may involve multiple stress-associated pathways [9,10,11]. More recently, tomentosin has been reported to modulate apoptosis, autophagy, and endoplasmic reticulum (ER) stress pathways in colorectal cancer cells, suggesting that its anticancer activity may involve multiple intracellular stress-response mechanisms [12]. However, the exact mechanisms of action of tomentosin in lung cancer cells do not appear to be fully understood.

Cellular protein homeostasis plays a critical role in the survival of cancer cells. Due to rapid proliferation, elevated metabolic demand, and chronic oxidative stress, tumor cells generate excessive amounts of damaged, misfolded, or short-lived proteins and therefore become highly dependent on efficient protein quality-control systems [13,14]. In this process, the removal of misfolded or damaged proteins is primarily achieved through the ubiquitin–proteasome system (UPS) and autophagy pathways. The balance between these two systems is crucial for the adaptation of tumor cells to stress conditions and has emerged as a potential therapeutic vulnerability in cancer [13,14,15].

In this context, p97/VCP, an ATP-dependent AAA-ATPase plays a central role in ERAD, extraction of ubiquitinated proteins, membrane trafficking, DNA damage responses, and autophagosome maturation [16,17]. p97/VCP overexpression in cancer cells has been associated with cell proliferation, invasion, metastasis, and treatment resistance, highlighting its importance as a potential therapeutic target [18]. Altered p97/VCP expression accompanied by impaired autophagy has also been reported in pathological human tissues, further supporting its role in cellular stress adaptation and protein clearance mechanisms [19].

SVIP, one of the adaptor proteins that interact with p97/VCP, can modulate protein degradation processes by regulating the intracellular localization and function of p97/VCP. The interaction of SVIP with p97/VCP is particularly important in ERAD-related degradation processes and lysosomal trafficking, thereby exerting decisive effects on proteostatic balance [20]. In addition, SVIP has been implicated in the regulation of steroidogenic pathways through modulation of ERAD machinery, suggesting a broader role in intracellular homeostasis [21]. In pancreatic cancer cells, the p97/VCP–SVIP system has also been associated with invasion and migration potential, indicating possible relevance to malignant behavior [22].

On the other hand, p62, which functions as a selective autophagy receptor, is an important adaptor protein that recognizes ubiquitinated proteins and directs them to the autophagic degradation pathway [23,24]. p62 is involved not only in autophagy but also in cellular signaling. By regulating critical pathways such as nuclear factor-κB (NF-κB) and nuclear factor erythroid 2-related factor 2 (Nrf2), it influences cell survival, inflammation, oxidative-stress responses, and chemoresistance. Changes in p62 accumulation or expression are closely associated with cancer progression and treatment response [25,26].

Although p97/VCP, SVIP, and p62 function at distinct stages of intracellular protein quality-control systems, they collectively contribute to the maintenance of proteostasis under stress conditions. Dysregulation of these proteins may critically influence cellular fate by modulating apoptosis, proliferation, and adaptive survival responses. To date, however, the effects of tomentosin on these proteostasis-associated proteins in lung cancer cells have not been clarified.

Cisplatin was selected as the combination partner because platinum-based chemotherapy remains an important component of NSCLC treatment, while its clinical effectiveness may be limited by treatment resistance and dose-related toxicity [2,3]. Accordingly, the combined treatment was included to determine whether concomitant exposure to tomentosin and cisplatin would produce a greater biological response than either agent alone and whether this response was accompanied by alterations in proteostasis-related proteins. The combination experiments were exploratory and were not designed to establish pharmacological synergy.

Therefore, this study aimed to evaluate the cytotoxic effects of tomentosin on human lung adenocarcinoma A549 cells and to compare their response with that of bronchial epithelium cells BEAS-2B. Additionally, the study aimed to evaluate the effects of tomentosin, alone and in combination with cisplatin, on apoptosis, cell cycle distribution, and the levels of the proteostasis-related proteins p97/VCP, SVIP, and p62 in A549 cells.

## 2. Materials and Methods

### 2.1. Plant Material and İsolation of Tomentosin

*D. viscosa* was collected from the city center of Hatay, Türkiye, in July 2025, and taxonomically identified by Fevzi Özgökçe. A voucher specimen was deposited in the Herbarium of Van Yüzüncü Yıl University under the accession number VANF 16286. Tomentosin was isolated from the leaves of *D. viscosa* according to the method previously described by Aydın et al. [27]. Briefly, dried and powdered leaf samples (100 g) were extracted with ethanol (500 mL) at room temperature by maceration, and the extraction procedure was repeated five times. The combined filtrates were concentrated under reduced pressure using a rotary evaporator, yielding 18 g of crude ethanol extract. The extract was subjected to silica gel column chromatography and monitored by thin-layer chromatography. Elution was performed using dichloromethane:acetone (90:10 and 98:2, *v*/*v*). Fractions containing tomentosin were combined and repeatedly purified. Following chromatographic purification, 510 mg of tomentosin was obtained, corresponding to an isolation yield of 2.83% (*w*/*w*) relative to the crude extract and 0.51% (*w*/*w*) relative to the initial dry plant material.

The identity of tomentosin was confirmed by ^1^H and ^13^C NMR analyses, and the obtained spectral data were consistent with those previously reported for tomentosin (Figure S1) [27]. The identity of the isolated compound was further supported by LC-MS/MS analysis. The molecular mass was found to be consistent with that expected for tomentosin, and the purity of the isolated compound was determined to be 89.9% based on LC-MS/MS analysis. The corresponding NMR (Figures S2 and S3) and LC-MS/MS (Figure S4) spectra are provided in the Supplementary Material.

### 2.2. Cell Culture

The human lung adenocarcinoma cell line A549, a widely used epithelial-like model of non-small cell lung cancer (NSCLC), and the bronchial epithelium cell line BEAS-2B were used in this study. Cells were obtained from The American Type Culture Collection (ATCC) and cultured under sterile conditions throughout the experiments. A549 and BEAS-2B cells were cultured in Dulbecco’s Modified Eagle Medium (DMEM) (Capricorn Scientific, Ebsdorfergrund, Germany) containing 10% fetal bovine serum (FBS) (Invitrogen, Carlsbad, CA, USA) and 1% penicillin–streptomycin (Gibco, Grand Island, NY, USA). Cells were maintained at 37 °C in a humidified atmosphere containing 5% CO_2_, and passaged when they reached 80–90% confluence.

### 2.3. WST-1 Cell Viability Assay

Tomentosin and cisplatin were dissolved in DMSO (Merck, Saint Louis, MO, USA) at stock concentrations of 8 mM and 500 µM, respectively. For experimental treatments, the stock solutions were diluted with appropriate culture media to prepare the working concentrations. A549 and BEAS-2B cells were seeded at appropriate densities (2 × 10^4^ cells/well) in 96-well plates and incubated for 24 h to allow cell adhesion to the surface. The cells were then treated with tomentosin (0, 5, 10, 25, 50, 100, and 200 µM) and cisplatin (0, 1, 2.5, 5, 10, 25, and 50 µM). After treatment, the cells were incubated for 24 h and 48 h. At the end of the incubation period, 10 µL of WST-1 reagent (Cayman Chemical, Ann Arbor, MI, USA, catalog no. 10008883) was added to each well, and the cells were incubated at 37 °C for 4 h. Following incubation, the absorbance of the resulting formazan product was measured at a wavelength of 450 nm using a microplate reader (Thermo Scientific Varioskan, Norristown, PA, USA). The percentage of cell viability was calculated by normalizing it to the control group. The IC_50_ values were derived from dose–response analysis using GraphPad Prism (v9.1.0) [28].

### 2.4. Annexin V Binding Assay for Apoptosis Detection

Flow cytometric analysis was performed to assess apoptotic, necrotic, and viable cell populations. A549 cells were seeded into 6-well plates (5 × 10^5^ cells/well) and incubated for 24 h to allow cell adhesion to the surface. A549 cells were treated with tomentosin (24.46 µM) and cisplatin (20.06 µM), corresponding to their respective 24 h IC_50_ concentrations, either alone or in combination, for 24 h. At the end of the incubation period, cells in the medium were collected, and cells adhering to the plate surface were separated with 0.25% trypsin-EDTA. All cells were combined, washed with phosphate-buffered saline (PBS), and the cell suspension was adjusted to 1 × 10^5^ cells/100 µL. Then, 5 µL of Annexin V-FITC and 5 µL of propidium iodide (PI) (BD Biosciences, Milpitas, CA, USA) along with 1X Annexin binding buffer were added to the cells. The cells were incubated at room temperature in the dark for 15 min. After incubation, cells were analysed using an ACEA NovoCyte flow cytometer (ACEA Biosciences Inc., San Diego, CA, USA). The data showed that both Annexin V and PI negative cells were considered viable. Annexin V positive and PI negative cells were classified as early apoptotic, cells positive for both Annexin V and PI were classified as late apoptotic, and PI positive and Annexin V negative cells were classified as necrotic.

### 2.5. Cell Cycle Analysis

Flow cytometric analysis was performed to determine cell-cycle distribution. A549 cells were plated in 6-well plates (5 × 10^5^ cells/well) and incubated for 24 h to allow cell adhesion to the surface. Cells were treated with IC_50_ concentrations of tomentosin and cisplatin and incubated for 24 h. At the end of the incubation period, cells were detached using 0.25% trypsin–EDTA, collected by centrifugation, and suspended in PBS. Cells were fixed in 70% ethanol at 4 °C for 20 min and then washed twice with PBS. For DNA staining, cells were treated with propidium iodide (PI), a fluorescent dye that intercalates into the major groove of double-stranded DNA. Since PI can also bind to double-stranded RNA, cells were treated with RNase A to improve the accuracy of DNA measurements. For this purpose, cells were incubated in a solution containing 40 µg/mL PI, 0.1% NP-40, and 20 µg/mL RNase A in the dark at 4 °C for 30 min. After incubation, the DNA content of the cells was analysed using a flow cytometer (ACEA Biosciences Inc., San Diego, CA, USA). Based on the obtained data, the distribution of cells in the G0/G1, S, and G2/M phases was determined, and the effects of tomentosin and cisplatin treatments on the cell cycle were evaluated.

### 2.6. Caspase-3/7 Activity Assay

To determine the ability of test compounds to induce apoptosis through the activation of caspase-3/7, A549 cells (5 × 10^5^ cells/well) were exposed to tomentosin, cisplatin either alone or in combination at their respective IC_50_ concentrations for 24 h in 6-well plates. Subsequently, the A549 cells were collected, washed with PBS, and then stained for the detection of caspase 3/7 activity for 30 min using a caspase-3/7 assay kit Tonbo Biosciences (Fisher Scientific, Pittsburgh, PA, USA) and NovoCyte D3000 flow cytometry instrument (ACEA Biosciences, Inc.).

### 2.7. Flow Cytometric Analysis of Intracellular Protein Expression

Flow cytometric analysis was performed to quantify intracellular protein levels. A549 cells were plated in 12-well plates (3 × 10^5^ cells/well) and incubated for 24 h to allow cell adhesion to the surface. The cells were treated with IC_50_ concentrations of tomentosin and cisplatin and incubated at 37 °C for 24 h. At the end of the incubation period, cells were collected and washed with PBS. Subsequently, cells were fixed with FCM fixation buffer for 15 min and permeabilized with FCM permeabilization buffer for 5 min. Following the washing steps, cells were stained with primary antibodies. For this purpose, p97/VCP (ab11433, Abcam, Cambridge, UK), p62 (ab91526, Abcam, UK), and SVIP (HPA039807; Sigma-Aldrich, USA) antibodies were used. Following primary antibody incubation, cells were incubated with the appropriate secondary antibody (Advansta, San Jose, CA, USA, R-05071) for 1 h. After incubation, cells were analysed using a NovoCyte D3000 flow cytometer (Agilent Technologies, Inc., Santa Clara, CA, USA). The obtained data were evaluated using NovoExpress 1.5.0 software (Agilent Technologies Inc.), and p97/VCP, p62, and SVIP protein expression was quantified as mean fluorescence intensity (MFI) and compared between control and treatment groups.

### 2.8. Statistical Analysis

The findings were analysed using GraphPad Prism version 9.1.0 (GraphPad Software, San Diego, CA, USA). All cell culture experiments were independently repeated three times (*n* = 3). Depending on the experimental design, comparisons were performed using one-way or two-way analysis of variance (ANOVA), followed by Tukey’s multiple-comparisons test. Data are presented as the mean ± standard deviation (SD), and *p* < 0.05 was considered statistically significant.

## 3. Results

### 3.1. Tomentosin Decreased Cell Viability on A549 Lung Cancer Cells in a Dose Dependent Manner

**Figure 1:**
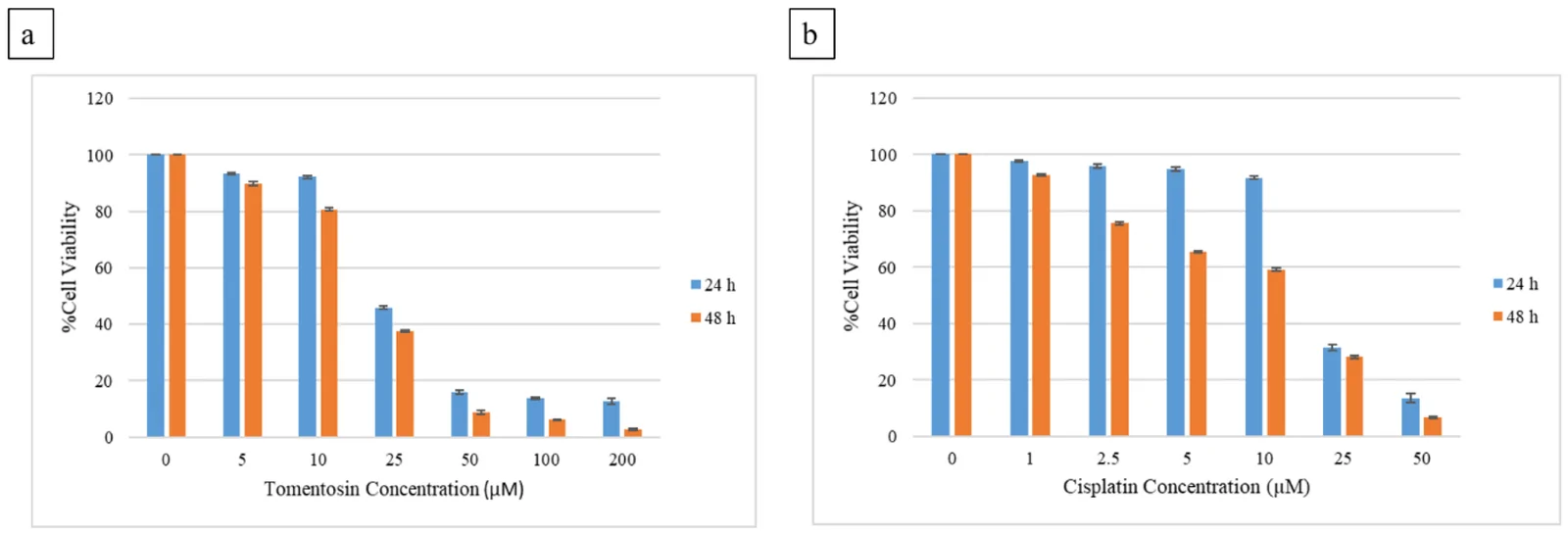
Effects of tomentosin and cisplatin on cell viability in A549 cells. (**a**) Dose-dependent effects of tomentosin on the viability of A549 lung cancer cells at 24 h and 48 h; (**b**) Dose-dependent effects of cisplatin on the viability of A549 cells at 24 h and 48 h. Cells were exposed to increasing concentrations of cisplatin (0–50 µM), and cell viability was determined by WST-1 assay. Data are presented as mean ± standard deviation (SD) of three independent experiments.

The cytotoxic effect of tomentosin on the A549 lung cancer cell line was evaluated using the WST-1 assay. The findings showed that tomentosin significantly reduced cell viability in A549 cells in a concentration- and time-dependent manner. In the 24 h treatment, a significant decrease in cell viability was observed with increasing tomentosin concentration. Cell viability values at concentrations of 5, 10, 25, 50, 100, and 200 µM were determined as 93.24 ± 0.34%, 92.24 ± 0.55%, 45.90 ± 0.70%, 15.83 ± 0.62%, 13.71 ± 0.51%, and 12.67 ± 0.95%, respectively (Figure 1a). Based on these data, the 24 h IC_50_ value of tomentosin was calculated as 24.46 ± 1.61 µM. It was observed that the cytotoxic effect became more pronounced after 48 h of treatment. For tomentosin, cell viability at concentrations of 5, 10, 25, 50, 100, and 200 µM was found to be 89.88 ± 0.67%, 80.75 ± 0.54%, 37.63 ± 0.44%, 8.79 ± 0.58%, 6.08 ± 0.12%, and 2.71 ± 0.50%, respectively (Figure 1a).

Cisplatin, used as a positive control, also showed time- and dose-dependent cytotoxic effects in A549 cells. In the 24 h treatment, cell viability at concentrations of 1, 2.5, 5, 10, 25, and 50 µM was found to be 97.69 ± 0.49%, 95.86 ± 0.65%, 94.90 ± 0.69%, 91.71 ± 0.51%, 31.53 ± 1.11%, and 13.55 ± 1.54%, respectively, and the IC_50_ value was determined as 20.06 ± 0.74 µM (Figure 1b). In the 48 h cisplatin treatment, a further decrease in cell viability was observed. At the same concentrations, cell viability values were calculated as 92.71 ± 0.50%, 75.63 ± 0.44%, 65.51 ± 0.41%, 59.21 ± 0.58%, 28.20 ± 0.59%, and 6.63 ± 0.45%, respectively (Figure 1b).

**Figure 2:**
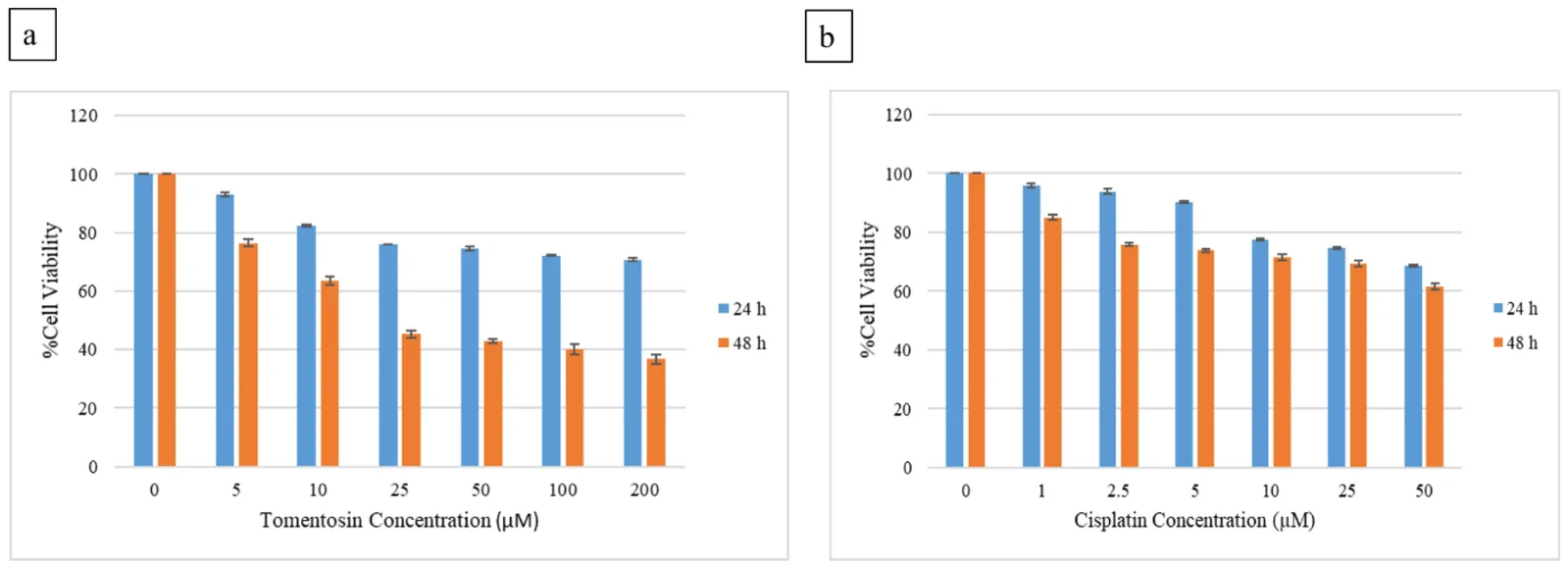
Effects of tomentosin and cisplatin on cell viability in BEAS-2B cells. (**a**) Effects of tomentosin on the viability of the bronchial epithelium cells BEAS-2B at 24 h and 48 h. (**b**) Effects of cisplatin on the viability of BEAS-2B cells at 24 h and 48 h. Cells were treated with increasing concentrations of cisplatin (0–50 µM), and cell viability was measured by WST-1 assay. Data are presented as mean ± standard deviation (SD) of three independent experiments.

Analyses performed on the bronchial epithelium cells BEAS-2B revealed that tomentosin showed limited cytotoxic effects after 24 h of treatment. Cell viability was found to be 92.98 ± 0.79%, 82.36 ± 0.45%, 75.93 ± 0.10%, 74.55 ± 0.78%, 72.19 ± 0.27%, and 70.73 ± 0.52% at concentrations of 5, 10, 25, 50, 100, and 200 µM, respectively, and the IC_50_ value was observed to be above 50 µM (Figure 2a). A more significant decrease in cell viability was observed in the 48 h treatment, with cell viability values at the same concentrations determined as 76.38 ± 1.30%, 63.54 ± 1.53%, 45.29 ± 1.19%, 42.92 ± 0.72%, 40.06 ± 1.71%, and 36.69 ± 1.73%, respectively (Figure 2a).

Similarly, following cisplatin treatment of BEAS-2B cells, cell viability values were 95.98 ± 0.79%, 93.94 ± 0.74%, 90.29 ± 0.41%, 77.50 ± 0.41%, 74.63 ± 0.45%, and 68.68 ± 0.48% after 24 h of treatment, and 85.04 ± 0.86%, 75.77 ± 0.56%, 73.79 ± 0.58%, 71.47 ± 1.05%, 69.48 ± 1.06%, and 61.54 ± 1.12% after 48 h of treatment, respectively. At both time points, the IC_50_ value was observed to be above 50 µM (Figure 2b).

At 24 h, the IC_50_ values were not reached in BEAS-2B cells within the tested concentration ranges and were therefore considered to be >200 µM for tomentosin and >50 µM for cisplatin. Based on the corresponding 24 h IC_50_ values in A549 cells, the selectivity index (SI), calculated as the IC_50_ in BEAS-2B cells divided by the IC_50_ in A549 cells, was estimated to be >8.18 for tomentosin and >2.49 for cisplatin. These values therefore represent lower-bound estimates rather than exact SI values. These findings indicate a comparatively greater selectivity of tomentosin toward A549 cancer cells under the investigated conditions.

Taken together, these findings indicate that tomentosin produced a greater reduction in viability in A549 cells than in BEAS-2B cells under the investigated experimental conditions. However, this differential response should not be interpreted as definitive evidence of tumor-selective cytotoxicity, as differences in the intrinsic growth characteristics of the two cell lines may influence viability-based comparisons.

### 3.2. Tomentosin Promoted Apoptosis in A549 Cells

**Figure 3:**
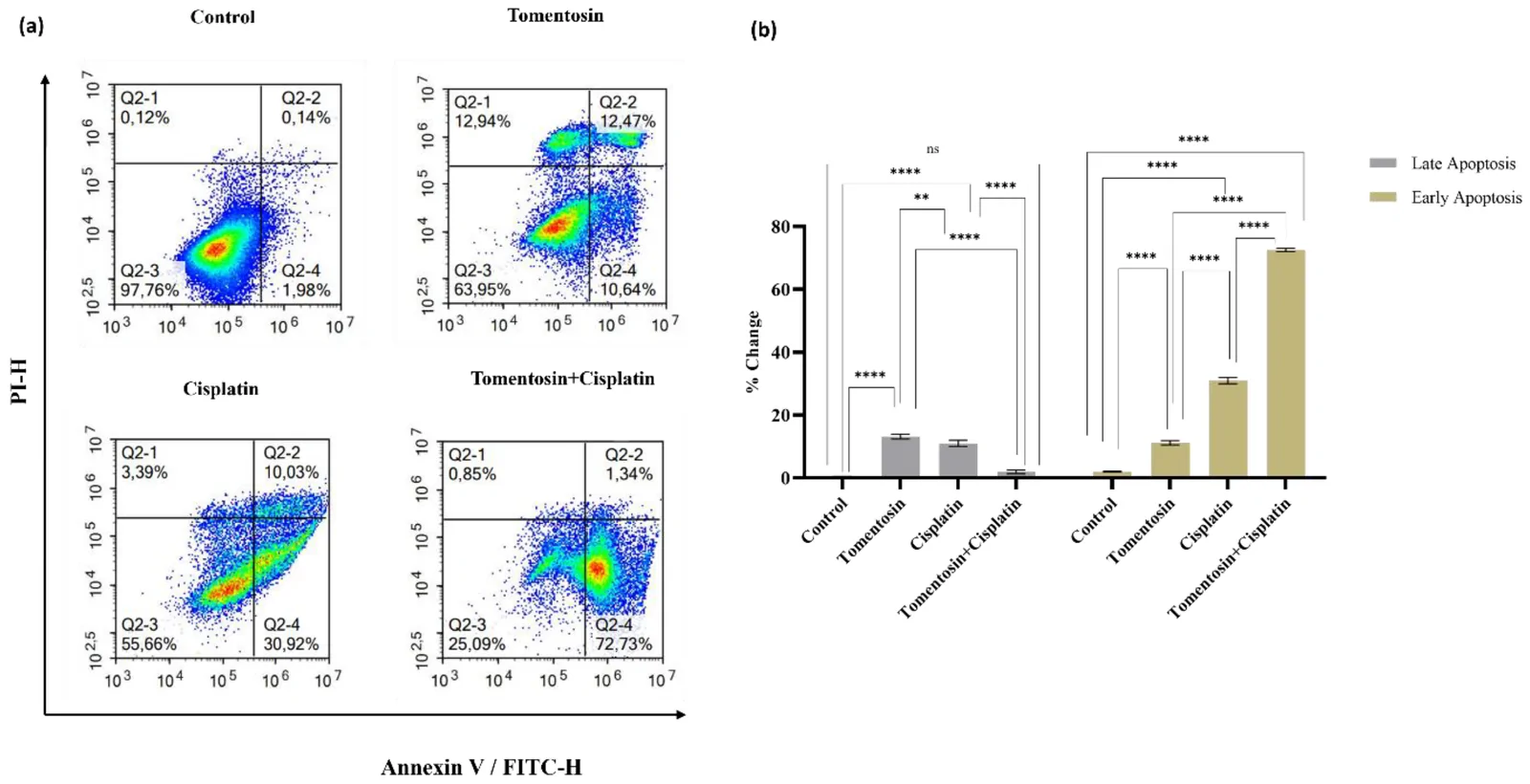
Effects of tomentosin and cisplatin on apoptosis in A549 cells. For the combined treatment, tomentosin (24.46 µM) and cisplatin (20.06 µM), corresponding to their respective 24 h IC_50_ values, were administered simultaneously for 24 h. (**a**) Representative Annexin V-FITC/PI flow cytometry dot plots and quantitative analysis of apoptotic cell populations in A549 lung cancer cells following treatment with tomentosin, cisplatin, and their combination for 24 h. In the dot plots, viable cells (Annexin V^−^/PI^−^) are located in the lower left quadrant (Q2-3), early apoptotic cells (Annexin V^+^/PI^−^) in the lower right quadrant (Q2-4), late apoptotic cells (Annexin V^+^/PI^+^) in the upper right quadrant (Q2-2), and necrotic cells (Annexin V^−^/PI^+^) in the upper left quadrant (Q2-1); (**b**) Control cells predominantly remained viable, whereas tomentosin and cisplatin treatments significantly increased both early and late apoptotic populations. Bar graphs represent the percentage distribution of early and late apoptotic cells. Data are presented as mean ± standard deviation (SD) of three independent experiments. Statistical significance was determined using two-way ANOVA followed by Tukey’s post hoc test (** *p* < 0.01, **** *p* < 0.0001; ns: not significant).

Apoptosis levels in A549 cells were assessed using Annexin V-FITC/PI flow cytometry. The findings showed that both tomentosin and cisplatin treatments significantly increased both early and late apoptosis rates. In the control group, the late apoptosis rate was 0.35 ± 0.18% and the early apoptosis rate was 2.06 ± 0.11%. In cells treated with tomentosin, the late apoptosis rate increased to 13.16 ± 0.77% and the early apoptosis rate increased to 11.21 ± 0.70%, and these increases were statistically significant compared to the control group (*p* < 0.0001) (Figure 3).

Following cisplatin administration, the rate of late apoptosis was determined to be 11.01 ± 0.99%, and the rate of early apoptosis was 30.97 ± 1.01%, showing a significant increase in both parameters compared to the control group (*p* < 0.0001). Furthermore, the effect of cisplatin on early apoptosis was more pronounced than that of tomentosin (*p* < 0.01) (Figure 3).

In the group treated with both tomentosin and cisplatin, the rate of early apoptosis reached a significantly higher value of 72.58 ± 0.47%, while the rate of late apoptosis was determined to be 1.95 ± 0.58%. These results indicate a marked predominance of the early apoptotic population following combined treatment. The rate of early apoptosis in the combination group was significantly higher compared to both the single-treatment groups and the control group (*p* < 0.0001) (Figure 3).

The necrotic cell population was 0.15 ± 0.001% in the control group, 11.95 ± 1.2% in the tomentosin-treated group, 3.5 ± 0.05% in the cisplatin-treated group, and 0.85 ± 0.002% in the combined-treatment group.

### 3.3. Tomentosin Induced Cell Cycle Arrest in A549 Lung Cancer Cells

**Figure 4:**
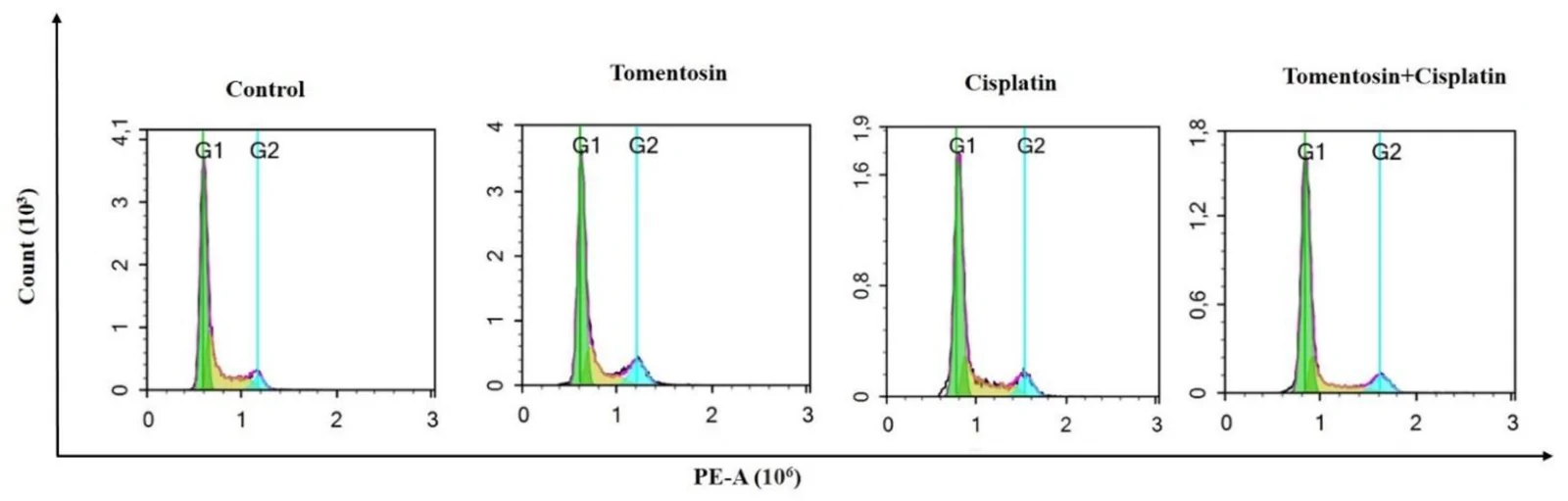
Representative histograms showing the distribution of A549 lung cancer cells across different phases of the cell cycle following treatment with tomentosin, cisplatin, and their combination. For the combined treatment, tomentosin (24.46 µM) and cisplatin (20.06 µM), corresponding to their respective 24 h IC_50_ values, were administered simultaneously for 24 h.

Cell cycle analysis results showed that tomentosin and cisplatin significantly affected the cell cycle distribution in A549 cells. In the control group, the vast majority of cells were in the G1 phase (55.84%), while 36.07% of the cells were in S phase and 7.40% were in G2/M phase, indicating that the cells were in an active proliferative state (Figure 4).

Significant changes in cell cycle distribution were observed after tomentosin administration. The proportion of cells in the G1 phase increased to 60.24%, while the S phase decreased to 23.82%, and the G2/M phase increased to 14.69%. These findings indicate that tomentosin suppresses the cell cycle progression, causing accumulation particularly in the G1 phase, and also arresting some cells in the G2/M phase (Figure 4).

In cells treated with cisplatin, the percentage of cells in the G1 phase increased to 61.70%, the S phase decreased to 27.50%, and the G2/M phase was determined to be 9.92%. These results indicate that cisplatin predominantly causes cell cycle arrest in the G1 phase (Figure 4).

In the group where tomentosin and cisplatin were administered together, the effects on the cell cycle were observed to be more pronounced. The percentage of cells in the G1 phase increased to 66.62%, the S phase decreased to 21.70%, and the G2/M phase was determined as 10.61%. This indicates that combined treatment more strongly suppresses cell-cycle progression and promotes cell accumulation, particularly in the G1 phase (Figure 4).

Overall, it was concluded that tomentosin suppresses proliferation in A549 cells by arresting the cell cycle in the G1 and partially in the G2/M phases, while cisplatin is more effective in the G1 phase, and combination therapy enhances this effect.

### 3.4. Tomentosin and Cisplatin Increased Caspase-3/7 Activity in A549 Cells

**Figure 5:**
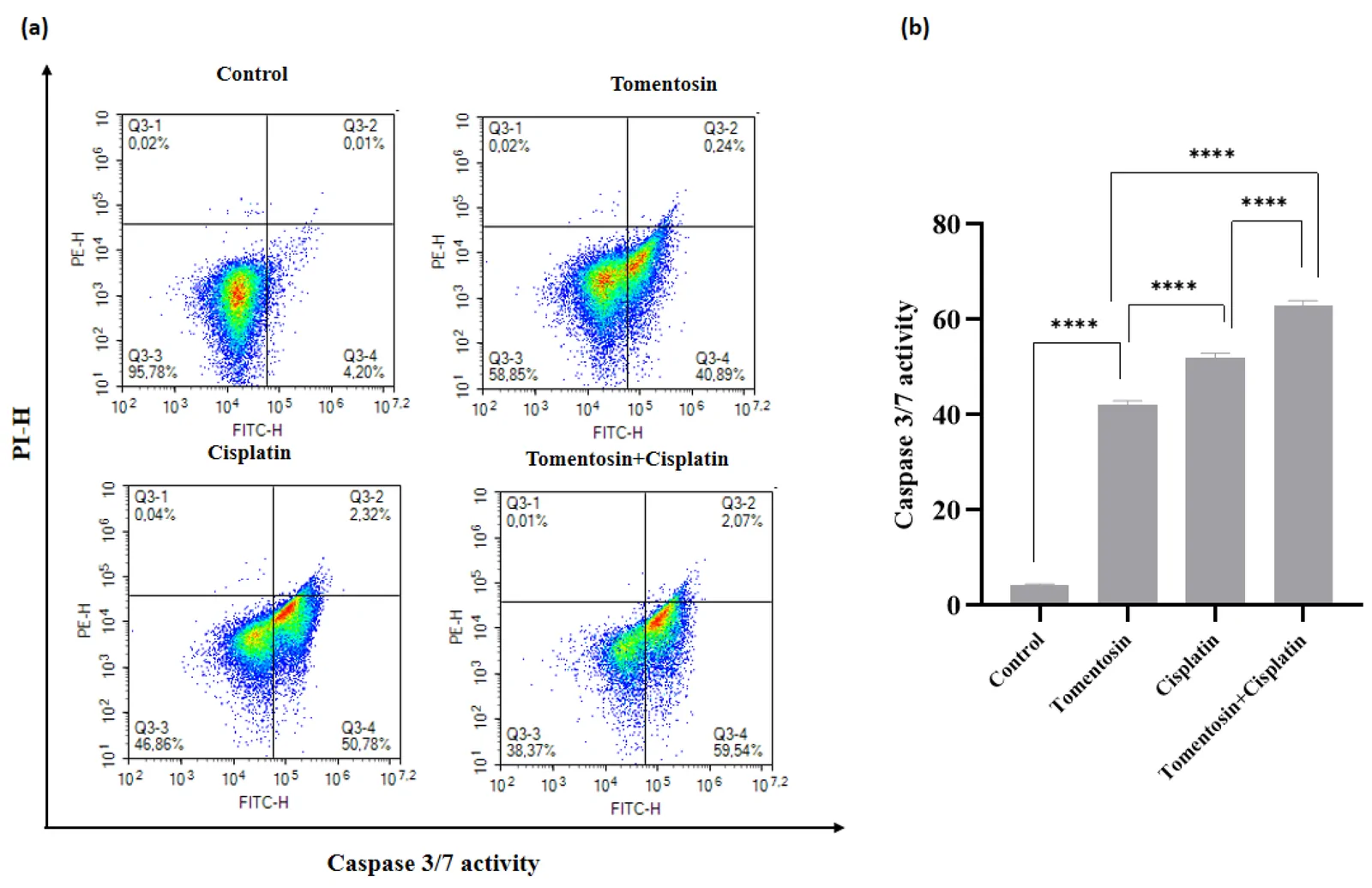
Effects of tomentosin and cisplatin on caspase-3/7 activity in A549 cells. For the combined treatment, tomentosin (24.46 µM) and cisplatin (20.06 µM), corresponding to their respective 24 h IC_50_ values, were administered simultaneously for 24 h (**a**) Representative flow cytometry dot plots showing caspase-3/7 activation in untreated control cells and cells treated with tomentosin, cisplatin, or their combination. (**b**) Quantitative analysis demonstrated that both tomentosin and cisplatin significantly increased caspase-3/7 activity compared with the control group. Combined treatment produced the highest caspase-3/7 activity and resulted in a significantly greater response than either single treatment. Data are presented as the mean ± standard deviation of three independent experiments. Statistical significance was determined using ordinary one-way ANOVA followed by Tukey’s multiple-comparison test (**** *p* < 0.0001).

Flow cytometric analysis was performed to evaluate caspase-3/7 activity in A549 cells following treatment with tomentosin, cisplatin, or their combination. Caspase-3/7 activity was 4.24 ± 0.25 in the control group and increased significantly to 42.04 ± 0.94 following tomentosin treatment (*p* < 0.0001). Cisplatin treatment further increased caspase-3/7 activity to 52.03 ± 0.95, which was significantly higher than both the control and tomentosin groups (*p* < 0.0001). The highest caspase-3/7 activity was observed in the combined-treatment group, reaching 63.00 ± 1.00. This value was significantly higher than those observed in the control, tomentosin, and cisplatin groups (*p* < 0.0001 for all comparisons) (Figure 5). These findings indicate that both tomentosin and cisplatin activated executioner caspases in A549 cells, while combined exposure was associated with a greater caspase-3/7 response than either treatment alone.

### 3.5. Tomentosin Modulates p97/VCP, p62 and SVIP Protein Levels in A549 Cells

Intracellular p97/VCP, SVIP, and p62 protein levels were quantified by flow cytometry and expressed as mean fluorescence intensity (MFI). The findings showed that tomentosin and cisplatin treatments had significant effects on the expression of proteostasis-related proteins. Specifically, significant changes were observed in the levels of proteins involved in cellular protein quality control mechanisms, depending on the treatment groups.

**Figure 6:**
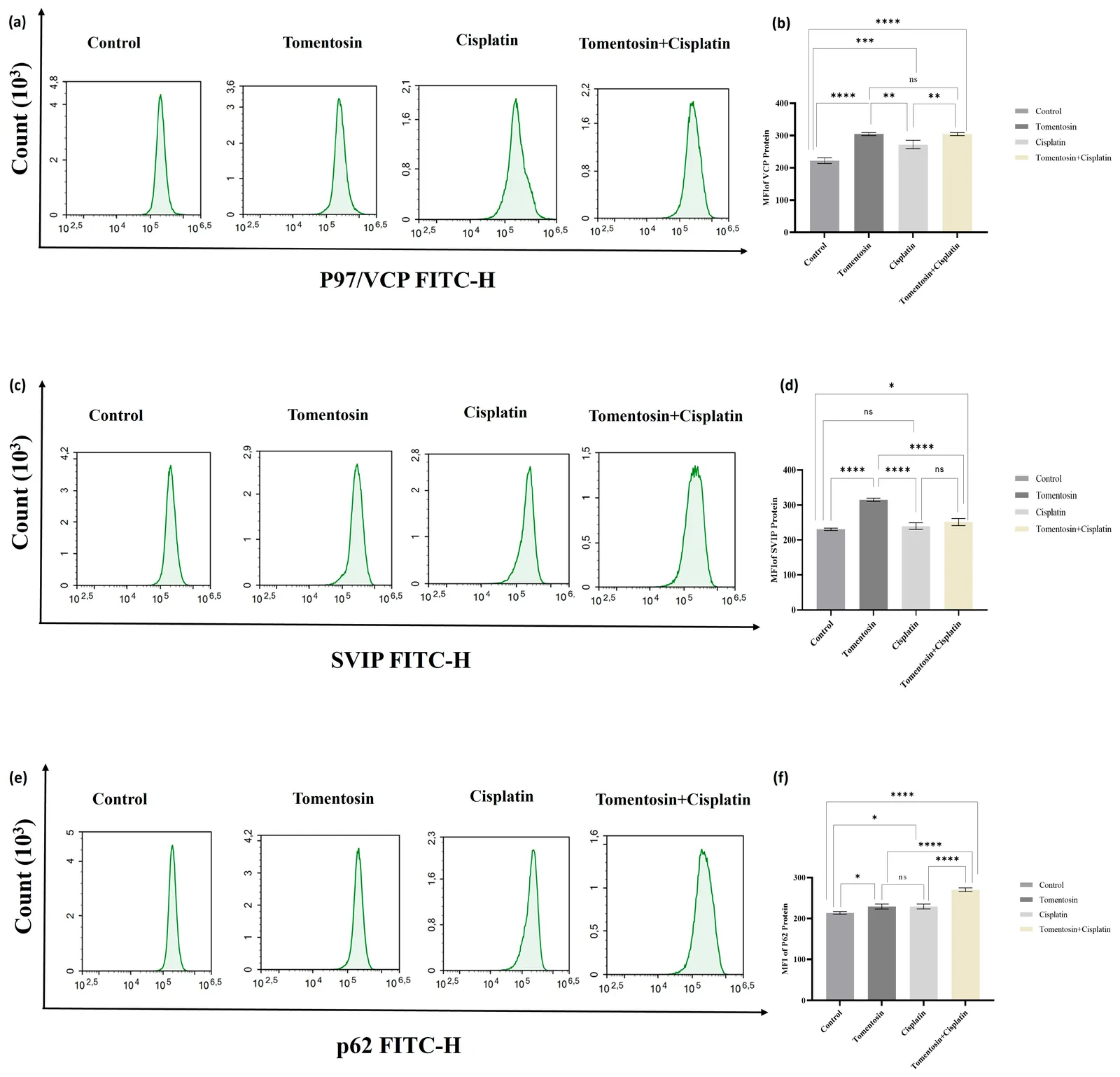
Effects of tomentosin and cisplatin on p97/VCP, SVIP, and p62 protein levels in A549 cells. A549 cells were treated for 24 h with tomentosin (24.46 µM), cisplatin (20.06 µM), or their combination, corresponding to their respective 24 h IC_50_ concentrations. Intracellular protein levels were determined by flow cytometry and expressed as mean fluorescence intensity (MFI). (**a**,**b**) p97/VCP; (**c**,**d**) SVIP; and (**e**,**f**) p62. Data are presented as mean ± SD from three independent experiments (n = 3). Statistical significance was determined using one-way ANOVA followed by Tukey’s multiple-comparison test (* *p* < 0.05, ** *p* < 0.01, *** *p* < 0.001, **** *p* < 0.0001; ns, not significant).

In the control group, the p97/VCP protein level was determined as 222.28 ± 8.07. In cells treated with tomentosin, this value increased to 304.44 ± 5.28, showing a statistically significant increase compared with the control group (*p* < 0.0001). In cells treated with cisplatin, the p97/VCP protein level was determined as 272.18 ± 13.52, which was also significantly higher than that of the control group (*p* < 0.001). In the group treated with both tomentosin and cisplatin, the p97/VCP level was 304.56 ± 4.09 and was significantly higher than that of the control group (*p* < 0.0001). Intergroup comparisons showed that the p97/VCP level was significantly higher in the tomentosin group than in the cisplatin group (*p* < 0.01). No significant difference was observed between the tomentosin and combination groups (ns), whereas the combination group showed a significantly higher p97/VCP level than the cisplatin group (*p* < 0.01) (Figure 6a,b).

In the control group, the SVIP protein level was determined as 231.17 ± 3.25. In cells treated with tomentosin, the SVIP level was found to be 315.17 ± 4.77, and this increase was statistically significant compared to both the control and cisplatin groups (*p* < 0.0001). Following cisplatin treatment, the SVIP level was determined to be 240.41 ± 9.36, and this value did not show a significant difference compared to the control group (ns). However, the combination group showed a significant difference compared to both cisplatin and tomentosin (*p* < 0.0001) (Figure 6c,d).

In the control group, the p62 protein level was determined as 213.71 ± 3.26. In cells treated with tomentosin, this value was found to be 229.30 ± 6.08, showing a statistically significant increase compared to the control group (*p* < 0.05). Following cisplatin treatment, the p62 level was determined as 229.33 ± 6.01, and this increase was also found to be significant compared to the control group (*p* < 0.05). However, no significant difference was found between the tomentosin and cisplatin groups (ns). In the group treated with both tomentosin and cisplatin, the p62 level was determined to be 270.13 ± 4.89, and this value was observed to be significantly higher than those in the control and single-treatment groups (*p* < 0.0001) (Figure 6e,f).

## 4. Discussion

The present study demonstrates that tomentosin exerts marked anticancer activity in A549 cells by reducing cell viability, promoting apoptosis, inducing cell cycle arrest, and modulating proteins involved in intracellular protein quality-control pathways. Importantly, these effects were further enhanced when tomentosin was combined with cisplatin, suggesting a potential chemosensitizing role. Given that platinum resistance and treatment-related toxicity remain major obstacles in non-small cell lung cancer (NSCLC), strategies capable of improving cisplatin efficacy are of considerable translational relevance [2,3].

A notable strength of the present study is the comparison between malignant A549 cells and the bronchial epithelial BEAS-2B cells. Tomentosin displayed comparatively lower cytotoxicity in BEAS-2B cells while exerting a pronounced dose- and time-dependent antiproliferative effect in A549 cells. However, differences in the intrinsic proliferative characteristics of these two cell lines should be considered when interpreting viability-based responses. The reported population doubling time is approximately 22 h for A549 cells, whereas BEAS-2B cells have been reported to exhibit a doubling time of approximately 26 h under standard DMEM-based culture conditions [29,30]. These differences in baseline proliferative activity may have contributed, at least in part, to the distinct viability responses observed following treatment. Therefore, the lower cytotoxic effect observed in BEAS-2B cells should not be interpreted as definitive evidence of tumor-selective cytotoxicity. Cancer cells typically operate under elevated basal oxidative, metabolic, and proteotoxic stress, rendering them more dependent on adaptive survival systems [13,14]; however, the extent to which these biological differences contribute to the differential response observed here cannot be determined from the present data.

Our findings are broadly consistent with previous reports describing the anticancer effects of tomentosin in different tumor models. In hepatocellular carcinoma cells, tomentosin induced apoptosis and cell cycle arrest through oxidative stress-associated mechanisms [9]. In osteosarcoma cells, it promoted intracellular reactive oxygen species generation and apoptotic cell death [10]. More recently, Çetinkaya et al. [12] demonstrated that tomentosin simultaneously modulates apoptosis, autophagy, and endoplasmic reticulum (ER) stress responses in colorectal cancer cells. The present study extends these observations to lung adenocarcinoma cells and further suggests that tomentosin may additionally interfere with intracellular proteostasis-associated pathways. Notably, a recent study evaluating tomentosin in A549 cells reported an IC_50_ value of 23.20 ± 1.26 µM after using the Alamar Blue assay [31]. This value is comparable to the 24 h IC_50_ of 24.46 ± 1.61 µM determined in the present study using the WST-1 assay, while a further reduction in cell viability was observed after 48 h in our experiments. Despite differences in the viability assay and experimental conditions, the comparable micromolar activity observed across the two studies supports the reproducibility of the antiproliferative effect of tomentosin in A549 cells.

Apoptosis analyses revealed that tomentosin significantly increased both early and late apoptotic cell populations. More importantly, combined tomentosin and cisplatin treatment resulted in a striking enrichment of early apoptotic cells compared with either single treatment. To the best of our knowledge, this is the first study to report that combined treatment with tomentosin and cisplatin was associated with an enhanced apoptotic response in A549 lung cancer cells compared with either treatment alone. This finding is particularly relevant because defective apoptotic signaling is a well-recognized mechanism contributing to platinum resistance in non-small-cell lung cancer (NSCLC) [3]. Therefore, tomentosin may enhance the apoptotic response of tumor cells to cisplatin. The predominance of the early apoptotic population in the combination group, together with the relatively low proportion of late apoptotic cells, may be related to the selected 24 h treatment period.The relatively low Annexin V−/PI+ necrotic population observed in the combination group compared with the tomentosin group should not be interpreted as evidence of reduced necrotic cell death. At the 24 h time point, the majority of affected cells in the combination group were detected within the Annexin V+/PI− early apoptotic population. Therefore, the observed distribution may reflect differences in the kinetics of cell-death progression. Since only a single time point was evaluated, time-course experiments would be required to determine whether these cells subsequently progress to late apoptosis or other cell-death states. Apoptosis is a dynamic process, and at this time point a substantial proportion of cells may have entered the early apoptotic phase without yet progressing to late apoptosis. Therefore, the observed distribution should be interpreted as a time-point-specific apoptotic response rather than evidence that combined treatment preferentially induces only early apoptosis. Additional time-course analyses would be required to determine whether these cells subsequently progress to late apoptosis.

The enhanced response observed with combined treatment is also consistent with the broader concept that natural compounds may improve responsiveness to platinum-based chemotherapy. Several phytochemicals, including curcumin, resveratrol, quercetin, and luteolin, have been reported to enhance the response to cisplatin by modulating oxidative stress, mitochondrial apoptosis, and pro-survival signaling pathways in lung cancer models [2,32]. Our data suggest that tomentosin may enhance the biological response to cisplatin while also affecting adaptive protein quality-control mechanisms.

Cell cycle analysis further supported the antiproliferative activity of tomentosin. Exposure to tomentosin resulted in a predominant accumulation of cells in the G1 phase, accompanied by a reduction in the S-phase fraction, indicating suppression of cell cycle progression. This effect was more pronounced in the combination group, suggesting a greater inhibitory effect on proliferative signaling. Previous studies have also reported tomentosin-mediated checkpoint arrest, although the dominant phase may vary according to tumor type, treatment duration, and cellular context [9,10]. Our findings indicate that G1 restriction may represent an important component of tomentosin responsiveness in lung adenocarcinoma cells.

Recent studies have shown that natural products can modulate autophagy by targeting a range of proteins directly or indirectly involved in this process [33]. In this context, it is important to consider that autophagy operates in close coordination with other proteostasis mechanisms, including ERAD and the ubiquitin–proteasome system. Therefore, natural compounds may exert their effects through the modulation of these interconnected pathways rather than acting on autophagy alone.

One of the notable molecular observations of the present study was the differential modulation of p97/VCP, SVIP, and p62 following treatment. Tomentosin significantly increased p97/VCP and SVIP expression, suggesting activation of adaptive protein quality-control responses. Combined treatment induced the highest p62 accumulation, consistent with impaired degradative capacity and enhanced proteotoxic stress. Rather than representing isolated molecular changes, these proteins collectively reflect the status of intracellular protein quality-control pathways, including ERAD, ubiquitin-dependent turnover, and autophagy. Cancer cells rely heavily on these systems to tolerate continuous metabolic and proteotoxic stress; therefore, disruption of proteostasis may critically influence treatment responsiveness [13,14].

p97/VCP is a multifunctional AAA+ ATPase that plays a central role in the extraction of ubiquitinated proteins, ERAD, membrane remodeling, and autophagic maturation [16,17]. In the present study, p97/VCP levels were significantly increased following both tomentosin and cisplatin treatment compared with the control group. Tomentosin treatment resulted in a significantly higher p97/VCP level than cisplatin treatment, whereas the p97/VCP level in the combined-treatment group was significantly higher than that observed with cisplatin but did not differ significantly from that observed with tomentosin alone. These findings indicate that tomentosin, either alone or in combination with cisplatin, is associated with a marked increase in p97/VCP levels. This increase may reflect an enhanced requirement for protein quality-control mechanisms in response to treatment-induced cellular stress. Thus, p97/VCP upregulation in our model may represent a compensatory cellular response aimed at maintaining intracellular protein homeostasis under pharmacological challenge rather than constitutive treatment resistance. This distinction is important, since chronic p97/VCP overexpression has been associated with aggressive tumor behavior and poor clinical outcome in several malignancies [18].

SVIP, an endogenous p97/VCP-interacting protein, regulates the localization and functional activity of p97/VCP and has been implicated in ERAD and lysosome-associated pathways [20]. Interestingly, SVIP expression was highest in the tomentosin-only group, whereas its induction was attenuated in the combination group. This finding may suggest that tomentosin alone triggers a regulated adaptive response involving SVIP-mediated fine control of p97/VCP-dependent proteostasis pathways. In contrast, under combined drug exposure, this regulatory response may not be sustained to the same extent, possibly reflecting exhaustion or destabilization of compensatory signaling under greater stress burden.

p62/SQSTM1 functions as a selective autophagy adaptor that links ubiquitinated cargo to autophagosomes and is commonly degraded during effective autophagic flux [23,24]. Utaipan et al. [34] reported that carbazole alkaloids obtained from *Murraya koenigii* increased the accumulation of p62. In the present study, p62 levels were elevated in all treatment groups, with the most pronounced accumulation observed in the combination group. Notably, not all anticancer studies report p62 accumulation after treatment. In some settings, decreased p62 levels are interpreted as enhanced autophagic flux, whereas increased p62 expression may indicate impaired degradation or excessive cargo burden [25]. Therefore, the marked p62 increase observed in our combination group may reflect treatment-specific overload of degradative systems rather than a universal autophagic response.

When interpreted collectively, the marked elevation of p97/VCP and p62, together with the lower SVIP response observed in the combination group compared with tomentosin treatment alone, suggests an altered proteostasis-associated response following combined exposure. In this context, elevated p97/VCP may reflect a compensatory response aimed at maintaining intracellular protein homeostasis, whereas p62 accumulation may indicate an increased cargo burden or incomplete resolution of treatment-induced cellular stress. In this context, p97/VCP upregulation may represent a compensatory response aimed at restoring intracellular protein homeostasis, whereas p62 accumulation may indicate an increased cargo burden or incomplete resolution of treatment-induced cellular stress. These alterations in proteostasis-related proteins may therefore be associated with the enhanced apoptotic response observed following combined treatment. However, because these findings are based on changes in protein expression, they should be regarded as evidence of proteostasis modulation rather than direct confirmation of the underlying functional mechanisms.

Overall, our findings suggest that tomentosin may enhance the biological effects of cisplatin by influencing apoptotic responses, cell-cycle progression, and intracellular protein quality-control pathways. Such multitarget activity may be relevant in tumor cells that adapt to single-pathway inhibition through compensatory mechanisms. However, the present results demonstrate only an enhanced biological response to the combined treatment under the investigated in vitro conditions.

The present study has several limitations. The mechanistic experiments were conducted only in A549 lung cancer cells, which limits the generalizability of the findings to different lung cancer subtypes. Although the bronchial epithelium cells BEAS-2B were included in the cytotoxicity experiments to evaluate the relative selectivity of tomentosin, validation in additional lung cancer cell lines is still required.

Intracellular p97/VCP, SVIP, and p62 expression was analysed by flow cytometry, which enables quantitative protein assessment at the single-cell level while accounting for treatment-associated cellular heterogeneity. Nevertheless, the observed alterations in these proteins are correlative and do not directly establish their functional involvement in the treatment response. In addition, changes in p62 levels alone are insufficient to determine the direction or activity of autophagic flux. Therefore, the findings related to autophagy should be interpreted cautiously and validated using additional molecular markers and functional autophagic flux assays.

A formal drug-interaction analysis was not performed in the present study, as the combination experiments were conducted using a single fixed-dose combination based on the respective IC_50_ values of tomentosin and cisplatin. Future studies incorporating multiple dose combinations, including lower concentrations of cisplatin, and formal combination-index analyses will be necessary to determine potential synergistic interactions and dose-reduction effects.

The proteostasis-related analyses were performed at a single treatment concentration and time point and were not validated by an independent protein-detection method such as Western blotting. Furthermore, functional perturbation experiments targeting p97/VCP, SVIP, or p62 were not performed. Therefore, the observed changes should be regarded as preliminary and correlative, and they do not establish whether alterations in these proteins represent a cause or a consequence of the cellular response to treatment. Future studies incorporating dose-dependent analyses, complementary protein-detection methods, and functional gain- or loss-of-function approaches will be required to clarify their mechanistic contribution.

Finally, the current findings are based exclusively on in vitro experiments and have not yet been confirmed in vivo. Further studies involving additional lung cancer cell lines, complementary protein-analysis methods, functional molecular approaches, and appropriate in vivo tumor models are required to clarify the underlying mechanisms and determine the translational potential of tomentosin.

## 5. Conclusions

The present findings suggest that tomentosin may serve as a potential adjunct anticancer compound by enhancing the biological effects of cisplatin in A549 cells. Tomentosin was also associated with alterations in proteostasis-related proteins; however, the functional significance of these changes requires further investigation. Additional studies using multiple lung cancer cell lines, formal drug-interaction analyses, and appropriate in vivo models are needed to determine its translational potential.

## Data Availability

The data supporting the findings of this study are available from the corresponding author upon reasonable request.

## Supplementary Materials

The following supporting information can be downloaded at https://www.mdpi.com/article/10.3390/cimb48101015/s1.

## Abbreviations

The following abbreviations are used in this manuscript:

p97/VCP	Valosin containing protein
SVIP	Small p97/VCP-interacting protein
p62	p62/SQSTM1
ERAD	Endoplasmic reticulum-associated degradation
NF-κB	Nuclear factor-κB
Nrf2	Nuclear factor erythroid 2-related factor 2
NSCLC	Non-small cell lung cancer

## References

[1] Sharma R. (2022). Mapping of global, regional and national incidence, mortality and mortality-to-incidence ratio of lung cancer in 2020 and 2050. Int. J. Clin. Oncol..

[2] Zhou L., Lu Y., Liu W., Wang S., Wang L., Zheng P., Zi G., Liu H., Liu W., Wei S. (2024). Drug conjugates for the treatment of lung cancer: From drug discovery to clinical practice. Exp. Hematol. Oncol..

[3] Herbst R.S., Morgensztern D., Boshoff C. (2018). The biology and management of non-small cell lung cancer. Nature.

[4] Sanci T.O., Terzi E., Oz-Bedir B.E., Gumustas M., Aydin T., Cakir A. (2024). Effect of herniarin on cell viability, cell cycle, and Erk protein levels in different stages of bladder cancer cells. Chem. Biodivers..

[5] Terzi E., Oz-Bedir B.E., Ercan E., Ozmedir-Sanci T., Jafarova S., Aydin T. (2025). β-Arbutin and Cisplatin: A combined approach to modulating apoptosis, cell viability, and migration in bladder cancer cells. Toxicol. Vitr..

[6] Terzi E., Ozdemir-Sanci T., Oz-Bedir B.E., Geneci F., Jafarova S., Aydin T. (2025). Comparison of the effects of β-Arbutin on the NF-κB pathway in two-dimensional (2D) and three-dimensional (3D) colorectal cancer cell lines. Chem.-Biol. Interact..

[7] Migheli R., Virdis P., Galleri G., Arru C., Lostia G., Coradduzza D., Muroni M.R., Pintore G., Podda L., Fozza C. (2022). Antineoplastic Properties by Proapoptotic Mechanisms Induction of *Inula viscosa* and Its Sesquiterpene Lactones Tomentosin and Inuviscolide. Biomedicines.

[8] El Omari N., El Menyiy N., Zengin G., Goh B.H., Gallo M., Montesano D., Naviglio D., Bouyahya A. (2021). Anticancer and Anti-Inflammatory Effects of Tomentosin: Cellular and Molecular Mechanisms. Separations.

[9] Yu S., Lee C., Ha S., Lee J., Jang K., Park S. (2021). Induction of cell cycle arrest and apoptosis by Tomentosin in hepatocellular carcinoma HepG2 and Huh7 cells. Hum. Exp. Toxicol..

[10] Lee C.M., Lee J., Nam M.J., Choi Y.S., Park S.-H. (2019). Tomentosin Displays Anti-Carcinogenic Effect in Human Osteosarcoma MG-63 Cells via the Induction of Intracellular Reactive Oxygen Species. Int. J. Mol. Sci..

[11] Güçlü E., Çınar Ayan İ., Dursun H.G., Vural H. (2022). Tomentosin induces apoptosis in pancreatic cancer cells through increasing reactive oxygen species and decreasing mitochondrial membrane potential. Toxicol. Vitr..

[12] Çetinkaya S., Güçlü E., Çınar Ayan İ., Vural H., Dursun H.G. (2025). A sesquiterpene lactone, Tomentosin, as a novel anticancer agent: Orchestrating apoptosis, autophagy, and ER stress in colorectal cancer. Naunyn-Schmiedeberg’s Arch. Pharmacol..

[13] Li Y., Li S., Wu H. (2022). Ubiquitination-Proteasome System (UPS) and Autophagy Two Main Protein Degradation Machineries in Response to Cell Stress. Cells.

[14] Pohl C., Dikic I. (2019). Cellular quality control by the ubiquitin-proteasome system and autophagy. Science.

[15] Zhang Y., Zhang J., Fu Z. (2022). Role of autophagy in lung diseases and ageing. Eur. Respir. Rev..

[16] Zhang S., Mao Y. (2020). AAA+ ATPases in Protein Degradation: Structures, Functions and Mechanisms. Biomolecules.

[17] Ferrari V., Cristofani R., Tedesco B., Crippa V., Chierichetti M., Casarotto E., Cozzi M., Mina F., Piccolella M., Galbiati M. (2022). Valosin Containing Protein (VCP): A Multistep Regulator of Autophagy. Int. J. Mol. Sci..

[18] Costantini S., Capone F., Polo A., Bagnara P., Budillon A. (2021). Valosin-Containing Protein (VCP)/p97: A Prognostic Biomarker and Therapeutic Target in Cancer. Int. J. Mol. Sci..

[19] Ozsoy A.Z., Cayli S., Sahin C., Ocakli S., Sanci T.O., Ilhan D.B. (2018). Altered expression of p97/Valosin containing protein and impaired autophagy in preeclamptic human placenta. Placenta.

[20] Wang Y., Ballar P., Zhong Y., Zhang X., Liu C., Zhang Y.J., Monteiro M.J., Li J., Fang S. (2011). SVIP Induces Localization of p97/VCP to the Plasma and Lysosomal Membranes and Regulates Autophagy. PLoS ONE.

[21] Ilhan R., Üner G., Yilmaz S., Atalay Sahar E., Cayli S., Erzurumlu Y., Gozen O., Ballar Kirmizibayrak P. (2022). Novel regulation mechanism of adrenal cortisol and DHEA biosynthesis via the endogen ERAD inhibitor small VCP-interacting protein. Sci. Rep..

[22] Alimoğullari E., Çayli S. (2024). The function of p97/Valosin-containing protein (VCP) and small VCP-interacting protein (SVIP) in invasion and migration of pancreatic cancer cells. Turk. J. Med. Sci..

[23] Lippai M., Lőw P. (2014). The Role of the Selective Adaptor p62 and Ubiquitin-Like Proteins in Autophagy. BioMed Res. Int..

[24] Johansen T., Lamark T. (2011). Selective autophagy mediated by autophagic adapter proteins. Autophagy.

[25] Hennig P., Fenini G., Di Filippo M., Karakaya T., Beer H.-D. (2021). The Pathways Underlying the Multiple Roles of p62 in Inflammation and Cancer. Biomedicines.

[26] Komatsu M., Kageyama S., Ichimura Y. (2012). p62/SQSTM1/A170: Physiology and pathology. Pharmacol. Res..

[27] Aydin T., Saglamtas R., Dogan B., Kostekci E., Durmus R., Cakir A. (2022). A new specific method for isolation of Tomentosin with a high yield from *Inula viscosa* (L.) and determination of its bioactivities. Phytochem. Anal..

[28] Terzi E., Sanci T.O., Bedir B.E.O., Ercan E., Bağcı E.R., Çevik U.A., Sezginer E.K. (2026). Thiazole- and 1,3,4-Thiadiazole-Based Hybrids: Synthesis and In Vitro Cytotoxicity Against Human Cancer Cell Lines. Chem. Biol. Drug Des..

[29] Bang S.H., Hwang S., Choi S.Y., Kim H.J., Kim J.H., Lee S.H., Lee J.W., Son K.H. (2025). Enhanced Ciliogenesis of Human Bronchial Epithelial Cells by Simulated Microgravity. Life.

[30] ATCC. A549 (CCL-185). https://www.atcc.org/products/ccl-185.

[31] Salvatore M.M., Masi M., Suleman M., Kalath H., Karousa M.M., Ayoub M.M., Shaito A.A., Andolfi A. (2026). In Vitro and In Silico Insights into the Antiproliferative Activity of Sesquiterpenes from *Dittrichia viscosa* (L.) Greuter. Int. J. Mol. Sci..

[32] Talib W.H., Alsayed A.R., Barakat M., Abu-Taha M.I., Mahmod A.I. (2021). Targeting Drug Chemo-Resistance in Cancer Using Natural Products. Biomedicines.

[33] Martelli A., Omrani M., Zarghooni M., Citi V., Brogi S., Calderone V., Sureda A., Lorzadeh S., da Silva Rosa S.C., Grabarek B.O. (2022). New Visions on Natural Products and Cancer Therapy: Autophagy and Related Regulatory Pathways. Cancers.

[34] Utaipan T., Athipornchai A., Suksamrarn A., Jirachotikoon C., Yuan X., Lertcanawanichakul M., Chunglok W. (2017). Carbazole alkaloids from Murraya koenigii trigger apoptosis and autophagic flux inhibition in human oral squamous cell carcinoma cells. J. Nat. Med..

