# Supplementary material for: Tomentosin Potentiates Cisplatin-Induced Apoptosis and Modulates Proteostasis-Related Proteins in A549 Lung Cancer Cells
Source: Curr Issues Mol Biol. 2026 Oct 1;48(10):1015. doi: 10.3390/cimb48101015 (PMC13703366; doi:10.3390/cimb48101015)
Supplement: Supplementary file 1 [file cimb-48-01015-s001.zip › cimb-4505394-supplementary.pdf]

Tomentosin Potentiates Cisplatin-İnduced Apoptosis and Modulates Proteostasis-Related Proteins in A549 Lung Cancer Cells

Hilal Nakkas [1,2], Beyza Ecem Oz-Bedir [3,4], Emine Terzi [3,4], Tuba Ozdemir-Sanci [1,4], Monica Angela Neblea [5,*] and Tuba Aydin [6]

[1] Department of Histology and Embryology, Faculty of Medicine, Ankara Yildirim Beyazit University, Ankara, Türkiye; hilalnakkas@aybu.edu.tr (H.N.); ozdemirsanci.tuba@aybu.edu.tr (T.O.-S.)
[2] Ankara City Hospital, Ankara Yildirim Beyazit University, 06800, Ankara, Türkiye
[3] Department of Medical Biology, Faculty of Medicine, Ankara Yildirim Beyazit University, 06800, Ankara, Türkiye; beyzaecemozbedir@aybu.edu.tr (B.E.O.-B.); eterzi@aybu.edu.tr (E.T.)
[4] Yenimahalle Training and Research Hospital, Ankara Yildirim Beyazit University, 06170, Ankara, Türkiye
[5] Department of Natural Sciences, Pitesti University Center, National University of Science and Technology Politechnica Bucharest, 110040, Pitesti, Romania
[6] Department of Pharmacognosy, Faculty of Pharmacy, Ağrı İbrahim Çeçen University, 04100, Agri, Türkiye; taydin@agri.edu.tr

* Correspondence: monica.neblea@upb.ro

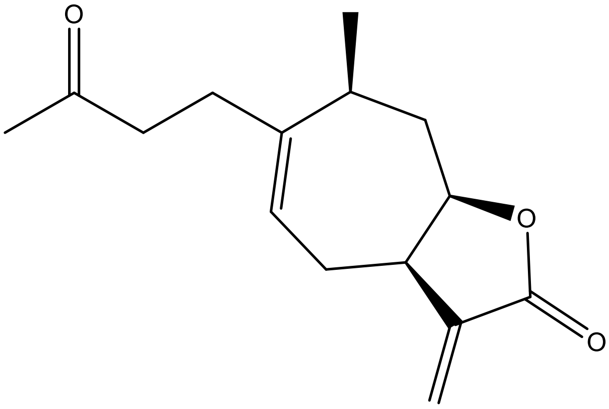

Figure S1. Chemical structure of tomentosin

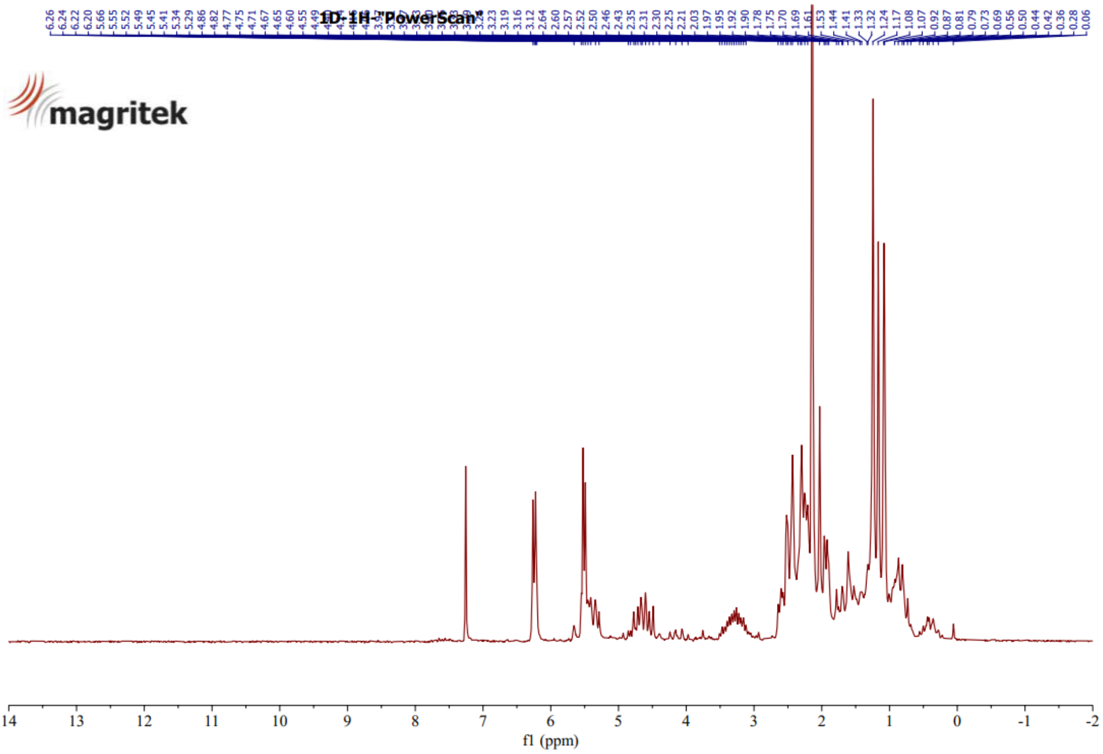

Figure S2. The ^{1}H NMR ($CDCl_3$, 80 MHz) spectrum of tomentosin

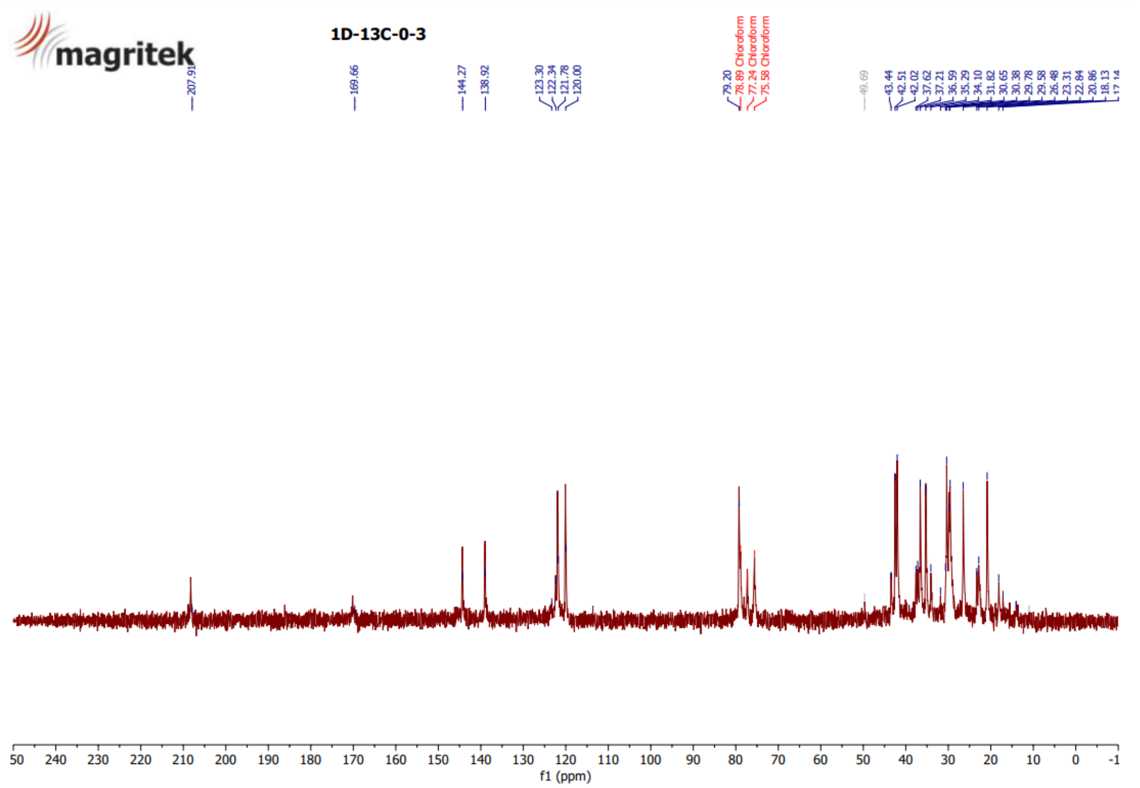

Figure S3. The ^{13}C NMR ($CDCl_3$, 20 MHz) spectrum of tomentosin

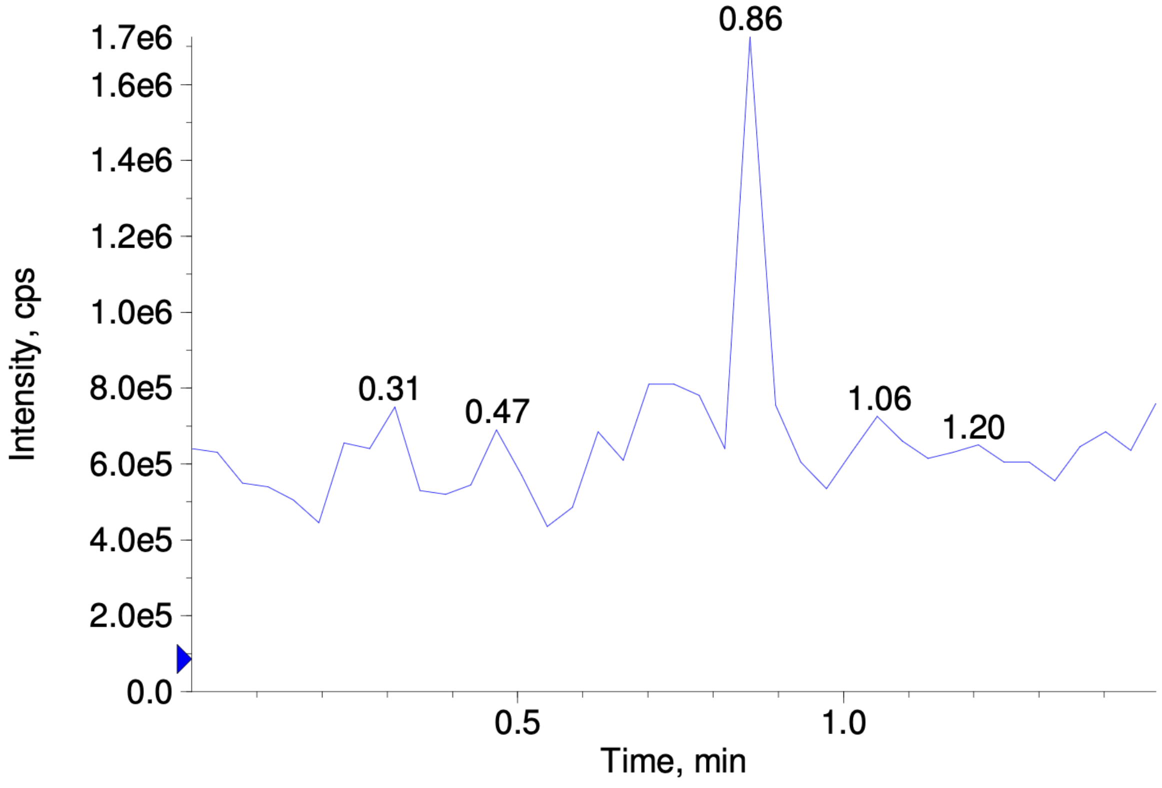

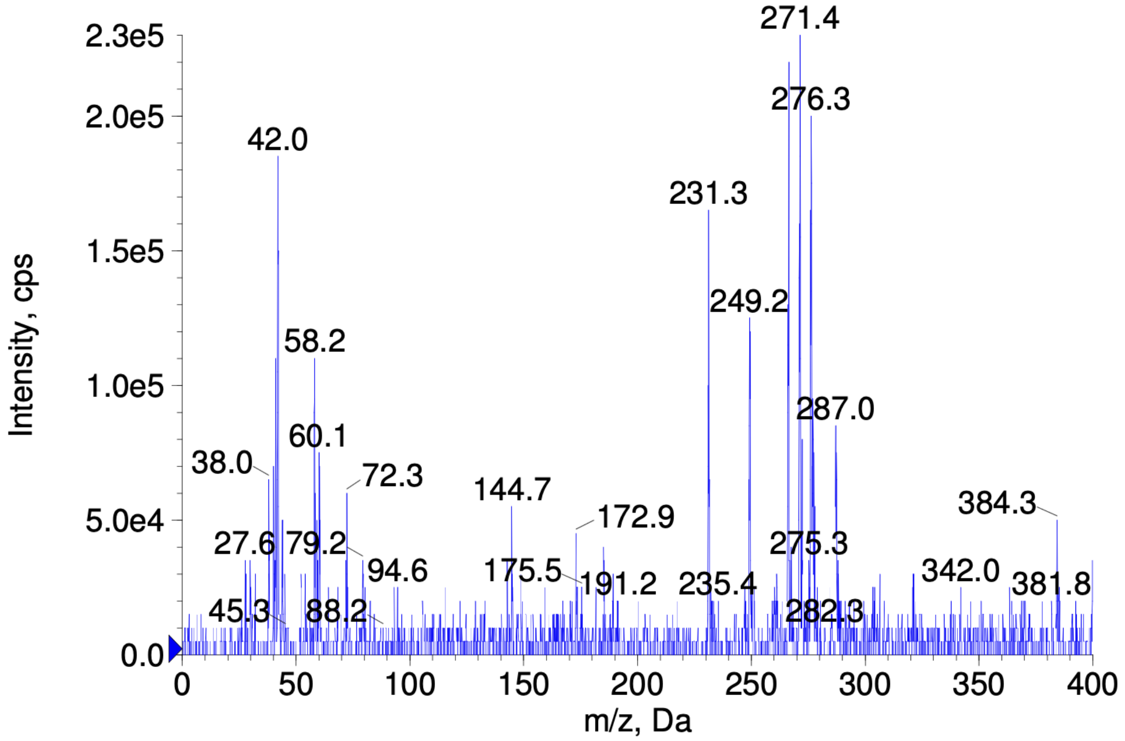

Figure S4. LC-MS/MS chromatogram and mass spectrum of isolated tomentosin. The chromatogram showed a major peak at a retention time (RT) of 0.86 min. LC-MS/MS spectrum exhibited the protonated molecular ion at m/z 249.2 $[M+H]^+$ together with the characteristic sodium and potassium adduct ions at m/z 271.4 $[M+Na]^+$ and m/z 287.0 $[M+K]^+$, respectively, confirming the identity of tomentosin.
